# Open lung approach versus standard protective strategies: Effects on driving pressure and ventilatory efficiency during anesthesia - A pilot, randomized controlled trial

**DOI:** 10.1371/journal.pone.0177399

**Published:** 2017-05-11

**Authors:** Carlos Ferrando, Fernando Suarez-Sipmann, Gerardo Tusman, Irene León, Esther Romero, Estefania Gracia, Ana Mugarra, Blanca Arocas, Natividad Pozo, Marina Soro, Francisco J. Belda

**Affiliations:** 1 Department of Anesthesiology and Critical Care, Hospital Clínico Universitario, Valencia, Spain; 2 CIBER de Enfermedades Respiratorias, Instituto de Salud Carlos III, Madrid, Spain; 3 Hedenstierna Laboratory, Department of Surgical Sciences, Uppsala University Hospital Uppsala, Sweden; 4 Department of Anesthesiology, Hospital Privado de Comunidad, Mar de Plata, Argentina; 5 INCLIVA Clinical Research Institute, Hospital Clínico Universitario, Valencia, Spain; Hospital Israelita Albert Einstein, BRAZIL

## Abstract

**Background:**

Low tidal volume (VT) during anesthesia minimizes lung injury but may be associated to a decrease in functional lung volume impairing lung mechanics and efficiency. Lung recruitment (RM) can restore lung volume but this may critically depend on the post-RM selected PEEP. This study was a randomized, two parallel arm, open study whose primary outcome was to compare the effects on driving pressure of adding a RM to low-VT ventilation, with or without an individualized post-RM PEEP in patients without known previous lung disease during anesthesia.

**Methods:**

Consecutive patients scheduled for major abdominal surgery were submitted to low-VT ventilation (6 ml·kg^-1^) and standard PEEP of 5 cmH_2_O (pre-RM, n = 36). After 30 min estabilization all patients received a RM and were randomly allocated to either continue with the same PEEP (RM-5 group, n = 18) or to an individualized open-lung PEEP (OL-PEEP) (Open Lung Approach, OLA group, n = 18) defined as the level resulting in maximal Cdyn during a decremental PEEP trial. We compared the effects on driving pressure and lung efficiency measured by volumetric capnography.

**Results:**

OL-PEEP was found at 8±2 cmH_2_O. 36 patients were included in the final analysis. When compared with pre-RM, OLA resulted in a 22% increase in compliance and a 28% decrease in driving pressure when compared to pre-RM. These parameters did not improve in the RM-5. The trend of the DP was significantly different between the OLA and RM-5 groups (p = 0.002). VDalv/VTalv was significantly lower in the OLA group after the RM (p = 0.035).

**Conclusions:**

Lung recruitment applied during low-VT ventilation improves driving pressure and lung efficiency only when applied as an open-lung strategy with an individualized PEEP in patients without lung diseases undergoing major abdominal surgery.

**Trial registration:**

ClinicalTrials.gov NCT02798133

## Introduction

Protective mechanical ventilation during anesthesia aims at minimizing lung injury and its inflammatory response, and has been associated to a decrease in postoperative pulmonary complications (PPCs) [1–3]. The currently recommended protective ventilation strategy is limited to the use of a low, more physiologic tidal volume (VT) [1]. A potential side effect of low-VT is the reduction of the functional volume of the lung manifested as lung collapse [4]. This increases lung heterogeneity and thus the driving pressure (DP), i.e the pressure gradient needed to generate a given VT calculated as plateau pressure minus PEEP that scales VT to the size of the functional lung volume [5]. Another potential consequence of lung collapse is the impairment in ventilatory efficiency [6].

Alternative intraoperative ventilatory strategies such as the open lung approach (OLA) that combine a lung recruitment maneuver (RM) with an individualized positive end-expiratory pressure (PEEP) level that prevents lung collapse i.e open-lung PEEP (OL-PEEP) have demonstrated favourable effects improving lung mechanics, ventilatory efficiency and oxygenation in patients with previously normal lung function [7–12]. Similarly, a recent study including ARDS patients reported a decrease in DP and improve in gas exchange during an OLA ventilation strategy when compared with standard low-VT ventilation [13]. Therefore, this strategy may result in a more global “protective strategy” with potential beneficial effects on PPCs [14]. Conversely, the effects of a fixed PEEP with or without RM are not clearly beneficial^1^, and according to data coming from post-hoc analysis including a large number of patients ventilated during anesthesia, appear to be protective only when associated to a decrease in driving pressure [15]. However, these studies did not investigate specific ventilatory interventions aimed at decreasing DP. To our knowledge there are no studies prospectively investigating the effects on DP of different ventilatory strategies applied in healthy patients during surgery.

Based on the above we hypothesized that an OLA strategy would decrease driving pressure and dead space more efficiently for a given VT when compared to a standard low-VT and PEEP ventilation and when compared to a RM strategy using a fixed standard post-RM PEEP during low-VT ventilation. The aim of this study was to compare the effects on driving pressure of adding a RM to low-VT ventilation, with or without an individualized post-RM PEEP setting in patients without previous lung disease during anesthesia.

## Methods

### Study design and participants

The study was performed at the Department of Anesthesiology and Post-surgical intensive care at the Hospital Clínico Universitario de Valencia, Spain from July 2014 to October 2014 and registered on 8 June 2016 at ClinicalTrials.gov with identification no.NCT02798133. Even though we fully comply with the current legislation and the study was approved by the ethics committee and was registered in clinicaltrial.org, although retrospectively, we are aware of the delayed registration of the study which occurred because the study’s data collection was conducted in 2014 and at that time we were not aware that study registration was mandatory before patient enrollment. The authors confirm that all ongoing and related trials for this intervention are registered. The study was approved by the Local Ethics for Clinical Research Committee on 05 May 2014 (Chairperson: Dr. Antonio Peláez) and written informed consent was obtained from all patients. The protocol details are available upon request to the corresponding author. The study was designed as a randomized two parallel arm open study. Eligible subjects were randomized 1:1 to one of the two treatment arms. For randomization a computer generated list of random numbers was used. The participants were assigned to the study groups by the sequential opening of numbered envelopes containing the randomization assignment. The study included consecutive patients with ASA physical status I-III undergoing elective major abdominal surgery including pancreatic-duodenectomy, gastrectomy and liver resection. Exclusion criteria were: age of <18 years, ASA IV, laparoscopic surgery and previous known respiratory disease.

### General procedures

Patients received routine monitoring including nasopharyngeal temperature, ECG, pulse oximetry, and invasive arterial pressure. The depth of anesthesia was assessed by the bi-spectral index (BIS vista, Medtronic), and cardiac index (CI) with the Pulsioflex monitor (Pulsion Medical System, GETINGE Group, Munich, Germany).

After 5 min of breathing 80% oxygen, anesthesia was induced with Fentanyl 5 μg kg^-1^, Propofol 2.5 mg·kg^-1^, and Rocuronium 0.6 mg·kg^-1^. Sevoflurane was administered to maintain a BIS between 40–50. Patients received a continuous infusion of remifentanyl 0.1–0.4 μg·kg^-1^·min^-2^. Crystalloid solutions were continuously infused at a rate of 3 ml·kg^-1^·h^-1^.

The patient’s lungs were ventilated with the FLOW-i anesthesia station (Maquet, GETINGE Group, Solna, Sweden) in a volume-controlled ventilation (VCV) mode as follows: VT of 6 ml·kg^-1^ predicted body weight (PBW), inspiratory-to-expiratory ratio of 1:2 with an end-inspiratory pause of 10%, PEEP 5 cmH_2_O, inspiratory fraction of oxygen (FIO_2_) of 0.5 and respiratory rate (RR) adjusted to maintain end-tidal CO_2_ (EtCO_2_) between 35–45 mmHg.

### Specific monitoring

Respiratory system mechanics, volumetric capnography (VCap), and SpO_2_ were continuously monitored with the NM3 monitor and recorded with the DataColl Software (Both Respironics/Philips, Wallingford, CT) during the study period.

Respiratory system mechanics measurements included VT, peak and plateau airway pressures (Paw and Pplat), driving pressure (Pplat—PEEP), dynamic compliance (Cdyn = VT/ Paw—PEEP) and expiratory airway resistance (Raw = peak pressure − PEEP/flow).

#### Volumetric capnography

Ventilatory efficiency was evaluated through the concept of dead space by using VCap as previously described in the literature [16,17]. The mainstream CO_2_ sensor was zeroed and placed at the airway opening. The following VCap parameters were automatically calculated as previously described [18].

1. VTCO_2_,br, the ml of CO_2_ eliminated within one breath, was measured by integrating the area under the curve (AUC) of VCap.
2. Mixed expired fraction of CO_2_ (FECO_2_) was calculated dividing VTCO_2_,br by the expired VT. From this value, mixed expired partial pressure of CO_2_ (PECO_2_) was obtained by its conversion to ATPS pressure units (multiplying FECO_2_ by barometric minus water pressure).
3. Physiologic dead space (VDphys) was calculated noninvasively using Bohr’s formula:
  $$\text{VDBohr}\left(\text{VD/VT}\right)=\left({{\text{PACO}}_{2}-\text{P}\bar{\text{e}}\text{CO}}_{\text{2}}\right)/{\text{PACO}}_{2}.$$
  where PACO_2_ is the mean alveolar partial pressure of CO_2_ measured at the midpoint of the alveolar plateau of phase III.
4. The alveolar dead space to alveolar tidal volume ratio (VDalv/VTalv) was calculated as:
  VDalv/VTalv=(VDPhys−VDaw)/VTalv.
  where VDPhys is the physiologic and VDaw the airway dead space.
5. The normalized slope of phase III of the capnogram, (SnIII) was calculated as previously described [18]:
  $$\text{SnIII}=\text{SIII}/{\text{FECO}}_{2}=\text{SIII}/\left({\text{VTCO}}_{2}\text{,br/VT}\right)$$
  Arterial blood gas (ABG) samples were obtained before and at the end of the study protocol (ABL 520 Radiometer, Copenhagen, Denmark).

### Experimental protocol

After 30 min stabilization following the induction of anesthesia and once the patient was positioned and surgery was started, preRM-5 was recorded over a ten minutes period. The data collected included respiratory system mechanics, VCap derived parameters, SpO_2_, ABG and hemodynamics.

**Fig 1:**
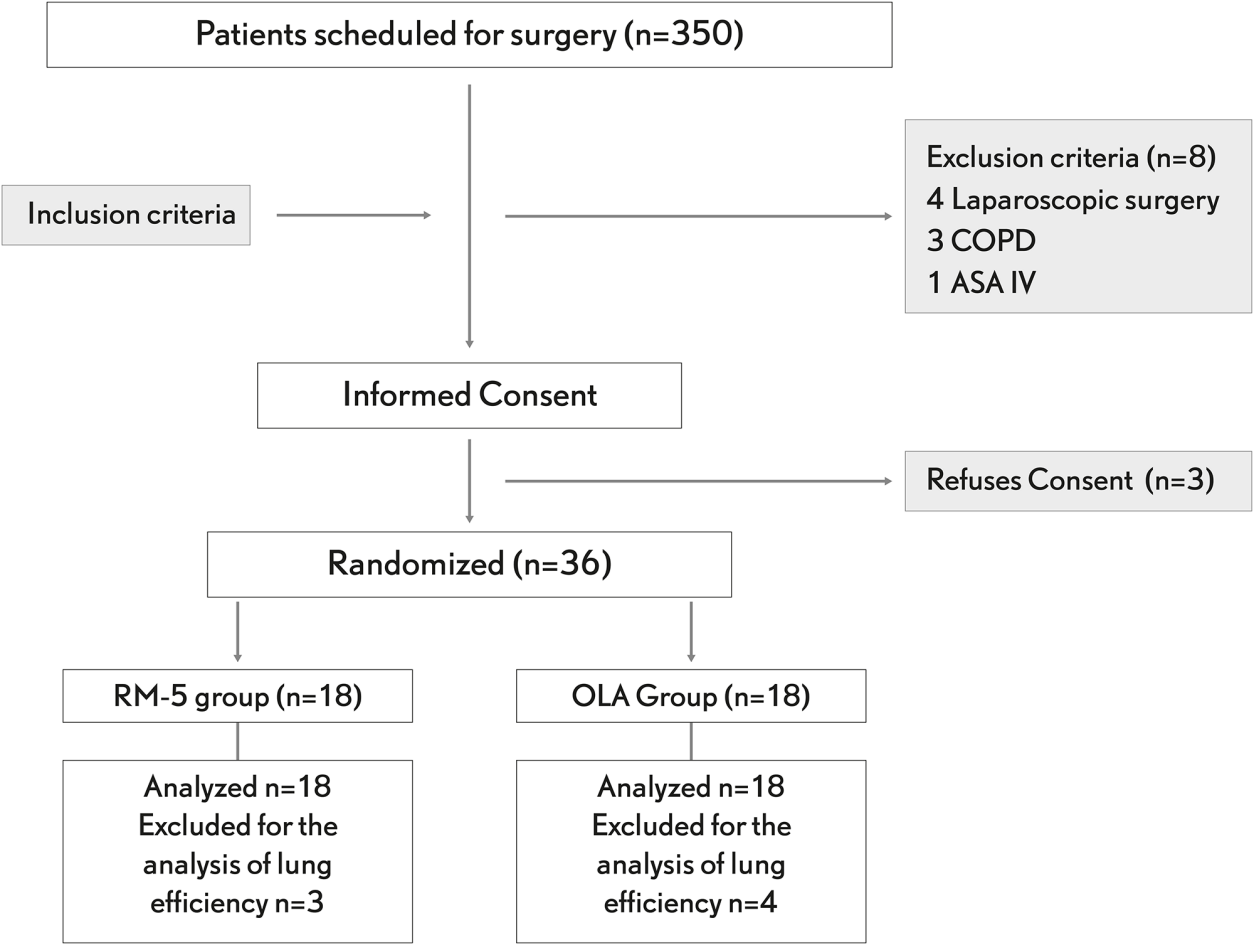
CONSORT flow diagram. COPD: chronic obstructive pulmonary disease, ASA: American Society of Anesthesiology physical status, RM: recruitment maneuver, OLA: open-lung approach.

Patients were then submitted to a recruitment maneuver and then randomly allocated to receive either OL-PEEP (OLA, n = 18) or fixed standard PEEP of 5 cmH_2_O (RM-5, n = 18) (Fig 1). The recruitment maneuver was performed as previously described [11]. In brief, after switching to a pressure controlled mode the initial PEEP level of 5 cmH_2_O was increased in 5 cmH_2_O steps each lasting ten breaths maintaining a fixed DP of 20 cmH_2_O until a recruitment inspiratory pressure of 40 cmH_2_O was reached which was then maintained for fifteen breaths. In the immediately following decremental PEEP titration trial, the ventilation mode was switched back to volume control with the same settings used during baseline but an initial PEEP of 20 cmH_2_O which was then reduced in steps of 2 cmH_2_O each maintained for 2 min [9]. The OL-PEEP was identified as the value corresponding to the highest Cdyn found during the decremental PEEP-titration trial. Finally, a new RM was performed to re-open collapsed alveoli and the OL-PEEP was applied and maintained during the rest of the study period. The postRM period started right after the respective postRM-PEEP was set in both groups and lasted for ten minutes.

In both periods, continuous measured data were obtained by averaging the final 5 min of recording. The discontinuous data were obtained at the end of the 5 min recording under stable patient condition. Any detected adverse event was registered in the study case report form.

### Statistical analysis

Due to the lack of data in the literature, our sample size was estimated based on previous own non-published data, it was estimated that a total of 32 patients were needed to detect at least a 10% difference in driving pressure after the PEEP adjustment, with a 5% significance level and 80% power. This figure was increased to 36 (10%) to compensate for possible dropouts (Fig 1). Primary outcome was the change in driving pressure from baseline, established thirty minutes after the induction of anesthesia to 5 minutes after the postRM condition. The secondary outcome was the change in ventilatory efficiency measured with volumetric capnography, including dead space and VCO2br, for the same period. Data were analyzed by an intention to treat approach. The study data is available at Open Science Framework. February 14. osf.io/3q2hj. Aditionally analyzed variables included respiratory and haemodinamic variables measured at the same time points as the primary outcome variables. The Kolgomorov-Smirnov with Lilliefor’s correction test was performed for variable normality and Levene’s test was used for homogeneity. Those variables with normal distribution were analyzed by using the independent t-Student test, the variables with non-normal distribution were analyzed by using the Kolmogorov-Smirnov nonparametric test. The trend of the different variables among the two groups was compared using repeated-measured analyses and a longitudinal model. The parameters are presented as mean (± SD). Statistical analysis was performed using the SPSS 20.0 software package (SPSS, Chicago, IL, USA). P-values < 0.05 were considered significant.

## Results

A total of thirty-six patients completed the study and were included in the analysis for the primary outcome. Seven patients were excluded for the analysis of lung efficiency because of defective CO_2_ recordings, related to different factors such as surgical manipulations and sensor dysfunction (Fig 1). The study was conducted in accordance to the original protocol. No adverse events related to the protocol were detected during the study time.

Demographic data of the patients are shown in Table 1. Included patients were evenly distributed according to ASA, ARISCAT score, age, sex, height and BMI.

**Table 1:** Participant characteristics.

	OLA	RM-5
ASA (II/III)	1/17	3/15
ARISCAT (moderate/high)	14/4	13/5
Age, yr	61 (11)	64 (8)
Women	9 (50)	10 (55)
Height, cm	168 (9)	166 (6)
Predicted Body weight, kg	59 (9)	60 (8)
BMI, kg·m^2^	24 (3)	26 (2)
Time of surgery, min	190 (75)	178 (86)
Liver resection	9 (50)	11 (61)
Pancreatic-duodenectomy	3 (17)	5 (28)
Gastrectomy	6 (33)	2 (11)

Data are presented as mean and standard deviation (SD) for continuous variables and n (%) for categorical variables. ASA = American association of anesthesiology physical status, ARISCAT score = predictive risk score (low, moderate, high) for postoperative pulmonary complication, BMI = body mass index. There were no significant differences between the groups (P>0.05).

**Fig 2:**
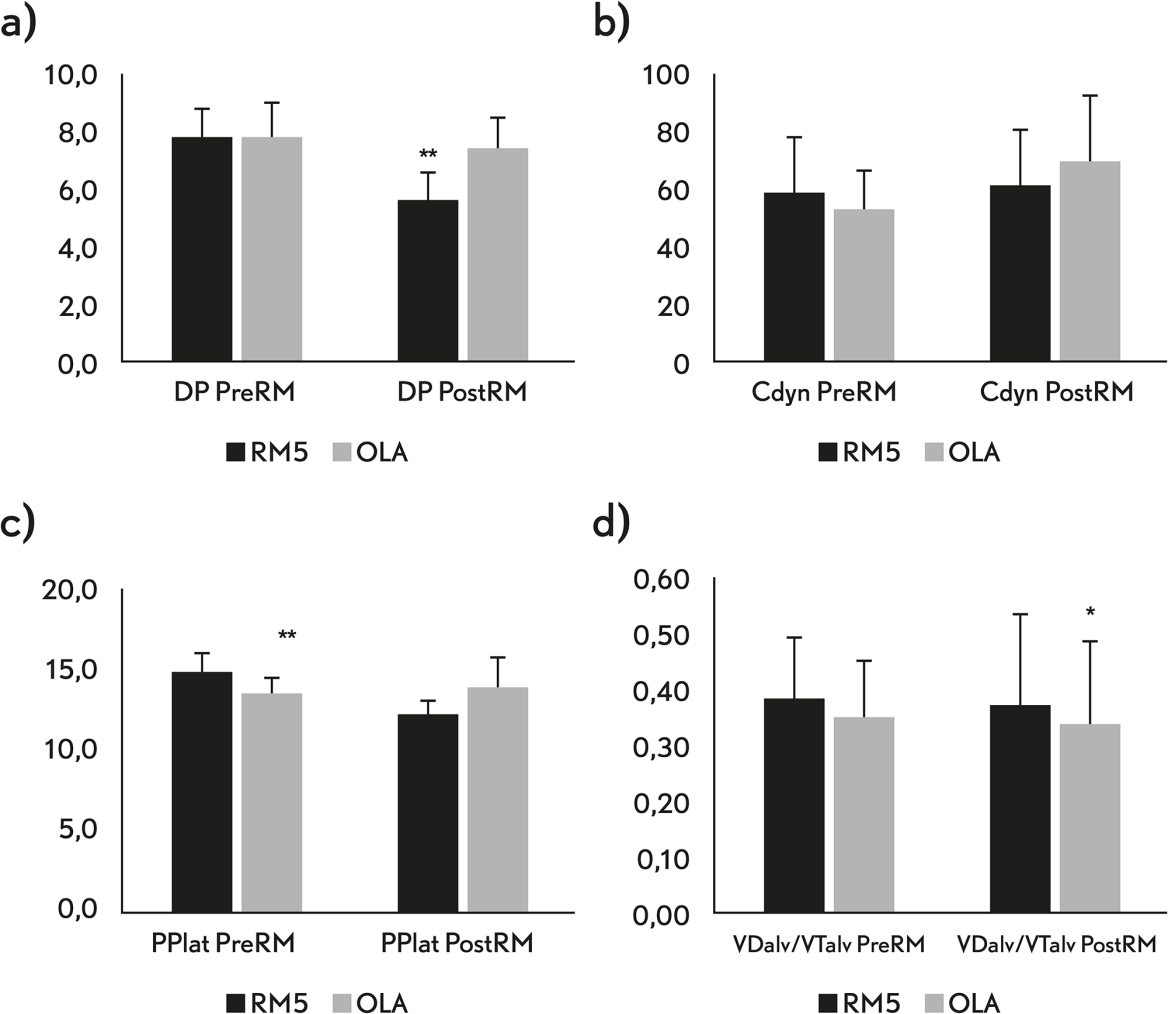
Respiratory system mechanics and ventilatory efficiency study variables. a) DP, driving pressure; b) Cdyn, respiratory system compliance; c) Pplat, plateau pressure; d) VTalv/VDalv, alveolar dead space to alveolar tidal volume. Values are shown as median and standard deviation. preRM and postRM represent values before and after the recruitment maneuver was performed. OLA represent values of the patients randomized to open-lung approach (OLA) and RM-5 represent values of the patients randomized to a post-RM PEEP of 5 cmH_2_O. * when P<0.05 and ** P<0.001.

The effects of the RM and PEEP setting on respiratory system mechanics and VCap (lung efficiency) are presented in Table 2 and Fig 2. The average OL-PEEP was found at 8±2 cmH_2_O with a range from 6 to 14 cmH_2_O. OLA resulted in a 22% increase in compliance and a 28% decrease in driving pressure when compared to pre-RM. These parameters did not improve in the RM-5. The trend of the DP was significantly different between the OLA and RM-5 groups (p = 0.002). When adjusting for the baseline DP as a covariate using a longitudinal model we found that DP postRM values where statistically different between the OLA and RM-5 groups (p<0.001). Although the VDalv/VTalv was significantly lower in the OLA group after the RM, the elimination of CO_2_ did not change after recruitment in neither of the groups.

**Table 2:** Ventilatory parameters, respiratory system mechanics and ventilatory efficiency variables.

Variables		Pre OLA	Pre RM-5	Pre RM-5 vs. Pre OLA p value	OLA	RM-5	RM-5 vs. OLA p value
**Ventilatory parameters** **n = 36**	**VT** **(ml)**	347±38	361±43	0.491	350 ± 36	361 ± 42	0.491
	**PEEP**, **cmH**_**2**_**O**	5,0 ± 0,0	5,0 ± 0,0	1.00	8,0 ± 2,3	5,0 ± 0,0	<0.001
	**RR, bpm**	14 ± 2	14 ± 1	0.667	14 ± 2	14 ± 1	0.667
**Respiratory system mechanics** **n = 36**	**DP** **(cmH**_**2**_**O)**	7.7 ± 1.0	7.7 ± 1.3	1.00	5.6 ± 1.0	7.4 ± 1.0	<0.001
	**Pplat** **(cmH**_**2**_**O)**	13.3 ±1.2	14.6 ± 1.2	<0.001	13.7 ± 1.9	12.2 ± 0.8	0.131
	**Cdyn (ml·cmH**_**2**_**O)**	53 ± 13	59 ± 19	0.945	68 ± 25	61 ± 19	0.903
	**Raw (cmH**_**2**_**O·l·s)**	11 ± 4	12 ± 4	0.314	11 ± 4	11 ± 3	0.272
**Ventilatory efficiency** **n = 29**	**VDBohr**	0.58 ± 0.11	0.59 ± 0.08	0.224	0.56 ± 0.11	0.56 ± 0.09	0.241
	**VDaw/VT**	0.36 ± 0.12	0.33 ± 0.06	0.314	0.32 ± 0.11	0.31 ± 0.06	0.771
	**VDalv/VTalv**	0.35 ± 0.16	0.38 ± 0.11	0.050	0.33 ± 0.15	0.37 ± 0.10	0.035
	**VTCO**_**2**_,**br (ml)**	8.29 ± 2.89	8.81 ± 2.02	0.861	8.20 ± 2.29	8.68 ± 1.90	0.963
	**FECO**_**2**_ **(%)**	4.06 ± 0.75	3.64 ± 0.48	0.271	3.97 ± 0.69	3.73 ± 0.82	0.421
	**SnIII** **(mmHg·ml)**	1.47 ± 0.99	1.19 ± 0.62	0.617	1.30 ± 1.13	1.02 ± 0.59	0.865

This table shows differences in respiratory system mechanics and ventilatory efficiency between the pre-OLA against pre-RM-5 and OLA against RM-5. Values are presented as mean and standard deviation (SD). VT = tidal volume, PEEP = positive end-expiratory pressure, RR = respiratory rate, DP = driving pressure, Pplat = plateau pressure, Cdyn = dynamic respiratory system compliance, Raw = airway resistance, VDaw = airway dead space to tidal volume, VDBohr = Bohr dead space, VDalv/VTalv = alveolar dead space normalized to alveolar VT, VCO_2_,br = amount of expired CO_2_ within one breath, FECO_2_: mixed expired CO_2_ in one breath, SnIII = slope of phase III normalized to VT.

It could not be found a correlation between the DP nor the change in DP (final- baseline values) and demographic variables (sex, age, height, BMI and PBW).

All patients remained hemodynamically stable during the protocol with a cardiac index (CI) between 2.3–3.5 ml·min^-1^·m^-2^, independently of the level of PEEP applied. CI never decreased more than 30% from its baseline value during the brief RM, fully recovering a few seconds after its conclusion. The PaO_2_/FIO_2_ baseline value was 386 ± 73 and increased to > 400 mmHg in all patients. The PaO_2_/FIO_2_ was higher in the OLA group, (473±51) vs. (437±64) but differences did not reach statistical significance (p = 0.07).

## Discussion

In this study we compared a conventional lung protective ventilation strategy with two lung recruitment strategies: the Open Lung Approach in which an individualized level of PEEP after RM is adjusted and a Lung Recruitment strategy using a fixed standard PEEP level after recruitment. We found that, even though lung recruitment per se could improve lung condition, OLA resulted in a better respiratory system mechanics, driving pressure and lung efficiency.

Atelectasis decrease functional residual capacity (FRC), increasing lung heterogeneity and negatively affecting driving pressure, ventilatory efficiency and gas exchange [19–22]. Atelectasis are considered a potential contributing factor for the development of PPCs [23] because they can promote tidal recruitment in the dependent and overdistension in the non-dependent lung [24]. The protective role of PEEP and its potential beneficial effects have not been clearly established, neither in patients during anesthesia [25] nor in ARDS patients [26,27]. This may be because PEEP per se does not restore FRC and just an arbitrarily set “higher” level of PEEP can contribute to overdistension and cause unwanted hemodynamic side effects, especially in heterogeneous lungs [28]. The results of our study suggest that individualizing PEEP may enhance its protective and minimizes its negative effects. Applying the same PEEP level after recruitment only resulted in a slight reduction in plateau pressure and physiological dead space. On the contrary OLA was associated to an increase in compliance and a decrease in driving pressure and physiological and alveolar dead spaces. These differences were significant despite the fact that OL-PEEP resulted on average only 3 cmH_2_O higher (i.e 5±0 vs 8±2 cmH_2_O). The individualized level of OL-PEEP found in this series of patients was, as expected, lower than the one found in other conditions such as in obese patients [9] or during one-lung ventilation [11], where higher mean levels of OL-PEEP (16 and 10 cmH_2_O, respectively) have been described. This suggests that it is not so much the absolute value of PEEP (high or low) but an adjusted level in an optimized lung condition what may makes the difference. In this context, the OL-PEEP seemed to be just enough to stabilize the recruited lung while avoiding overdistension and hemodynamic side effects when combined with low tidal volume ventilation. Following the same reasoning PEEP 5 cmH_2_O was just insufficient to stabilize the lung in most of the studied patients so that recruitment per se did not improve the lung condition as compared with pre-RM.

To our knowledge this is the first study comparing the effects of an OLA strategy using an OL-PEEP with a fixed standard PEEP with and without lung recruitment on driving pressure and ventilation efficiency in healthy patients undergoing surgery. Our results are in agreement with those obtained by our group using a similar OLA strategy during one lung ventilation. In this study we found that OLA resulted in better compliance and PaO_2_/FIO_2_ when compared with lung recruitment and fixed standard 5 cmH_2_O of PEEP [11]. In the same line, Kacmarek et al. [13] obtained a significant reduction in driving pressure when applying an OLA strategy in ARDS patients when compared with the conventional ARDSnet low tidal volume ventilation where PEEP was adjusted according to the PEEP/FIO_2_ table.

Another interesting finding of our study was the increased lung efficiency seen in the OLA group. Although recruitment decreased physiological and alveolar dead space with both post-RM PEEP settings as previously described [9,29,30], the trend in alveolar dead space was significantly better for the OLA group. Even though differences were small, this finding together with the higher Cdyn and lower DP obtained strongly suggests less functional overdistention despite a higher level of PEEP and a slightly higher plateau pressure during OLA. This could be explained by the maintenance of a more homogeneous collapse-free lung and the resulting lower driving pressure inducing less tidal strain [28]. Other VCap variables related to lung efficiency such as VTCO_2_br and FECO_2_ did not improve after recruitment independent from the PEEP setting.

### Clinical implications

Since we evaluated patients with normal lungs, and with a minimal amount of lung collapse, the observed differences in the studied parameters were as expected rather small. Nevertheless, we believe that they may have clinical relevance. A recent meta-analysis including more than 2000 patients studied during anesthesia found an association between each cmH_2_O of increased driving pressure and the risk of developing PPCs with and odds ratio of 1.16 [15]. A similar association was found by Ladha et al [31] in a registry study including 69.265 non-cardiac surgery patients. Also, recently Amato et al, found that driving pressure was the single most important independent predictor of mortality in ARDS patients [32]. Putting this information together, it seems that aiming ventilation at reducing DP while minimizing lung collapse and overdistension, maintaining ventilatory efficiency seems a desirable clinical target in terms of lung protection. In this respect an OLA strategy has the potential to enhance lung protection and decrease the risk for PPCs beyond the mere limitation of tidal volume or the use of an arbitrary fixed PEEP level even in healthy patients. It is likely that the benefits of an OLA strategy in patients with larger amount of anesthesia-induced atelectasis such as obese patients and patients submitted to laparoscopic or thoracic surgery, or those with pathological lungs are of greater clinical relevance but this remains to be established [7,8].

### Limitations

Our study has several limitations. This was a pilot physiological study and beyond the small number of patients, the estimated sample size based on non-published data and the relative small differences observed, the time spent on each ventilatory condition was relatively short. Although we describe a reduction in driving pressure, the response of the PEEP preventing a potential time-dependent alveolar re-collapse was not evaluated and therefore the sustained recruitment and minimization of DP was not assessed. Also, we do not provide any data on inflammatory markers or on post-operative outcomes. Therefore, the potential protective benefit of the OLA group remains speculative and limited to the study period. However, as mentioned, recent compelling evidence supports the benefits of reducing driving pressure [15,31,32].

### Conclusions

The use of an intraoperative OLA ventilation strategy results in better respiratory system mechanics, lower driving pressure and improved ventilatory efficiency when compared with a conventional low tidal volume ventilation strategy and fixed standard PEEP of 5 cmH_2_O, or with a RM strategy without an individualized post-RM PEEP titration. These results suggest that an OLA strategy may confer an enhanced lung protection even in this population of non-obese patients without lung disease undergoing major open abdominal surgery. Although these data support the use of lung recruitment with an individualized PEEP adjustment, healthy patients undergoing elective abdominal surgery are not likely to experience clinically significant lung injury and whether this will translate in less post-operative complications or improved outcomes in low or higher risk patients must be tested in future clinical studies.

## Supporting information

S1 CONSORT Checklist (DOC)

S1 File Original protocol. (DOCX)
