# Supplementary material for: Open lung approach versus standard protective strategies: Effects on driving pressure and ventilatory efficiency during anesthesia - A pilot, randomized controlled trial
Source: PLoS One. 2017 May 11;12(5):e0177399. doi: 10.1371/journal.pone.0177399 (PMC5426745; doi:10.1371/journal.pone.0177399)
Supplement: S1 File — (DOCX) [file pone.0177399.s002.docx]

**Study Protocol**

**Title: Efectos de la PEEP individualizada o estándar sobre la mecánica y eficiencia respiratoria.**

**Title: Effects of the individualized versus standard PEEP on respiratory mechanics and effiency.**

**Methods**

The study will be performed at the Department of Anesthesiology and Critical Care at the Hospital Clínico Universitario de Valencia, after the approval by the Local Ethics Committee. Written informed consent will be obtained from all patients. The recruitment of patients is planned to be of 6 months duration after the approval.

The study is designed as a randomized two parallel arm open study. Eligible subjects will be randomized 1:1 to one of the two treatment arms. For randomization a computer generated list of random numbers will be used. The participants will be assigned to the study groups by the sequential opening of numbered envelopes containing the randomization assignment. The study includes consecutive:

Inclusion criteria:

Patients with ASA physical status I-III undergoing elective pancreaticoduodenectomy and liver resection.

Exclusion criteria:

Age of <18 years, ASA more than III, laparoscopic surgery and patients with respiratory disease.

**2.4.- Sample size (n).**

The sample size needed to be included in the study was calculated by the statistical program GPower 3.1.5.

The “n” was calculated based in the primary outcome which is the behavior in driving pressure in 30 consecutives patients after individualized or standardized PEEP measured in a previous trial in patients undergoing one-lung ventilation. Based in the results of Ferrando et al (Anesth Analg 2014) Following measurements of the differences magnitude. (effects of the sample size) of 0.25^[[1]](#footnote-1)^; level of significance α = 0,05^[[2]](#footnote-2)^; power 1-β =95%^[[3]](#footnote-3)^;

[4] *-- Thursday, January 03, 204 -- 18:13:20*

**F tests -** ANOVA: Repeated measures, within factors

**Analysis:** A priori: Compute required sample size

**Input:** Effect size f = 0.32

α err prob = 0.05

Power (1-β err prob) = 0.95

Number of groups = 1

Number of measurements = 4

Corr among rep measures = 0.5

Nonsphericity correction ε = 1

**Output:** Noncentrality parameter λ = 18.0000000

Critical F = 2.6911329

Numerator df = 3.0000000

Denominator df = 105

Total sample size = 36

Actual power = 0.9519863

*General procedures*

Patients will be monitored for nasopharyngeal temperature, ECG, pulse oximetry, and invasive arterial pressure using the GE Aisys Carestation™ monitor. The depth of anesthesia will be monitored with the bispectral index (BIS vista, Aspect Medical Systems, The Netherlands), and cardiac index (CI) with the Pulsioflex monitor (Pulsion Medical System AG, Munich, Germany)

Prior to anesthesia induction, a thoracic epidural catheter (Tuhoy; Braun Laboratories, Melsungen AG, Germany) will be placed at T6 to T10, and 3 mL of bupivacaine 0.25% with epinephrine will be administered. After 5 min of breathing 0.8 oxygen, anesthesia will be induced with fentanyl 5 µg kg^-1^, propofol 2.5 mg kg^-1^, and rocuronium 0.6 mg kg^-1^. Sevoflurane will be administered to maintain a BIS between 40–50. Patients will received a continuous infusion of remifentanil 0.1–0.4 µg kg^-1^ min^-1^. Crystalloid solutions will be continuously infused at a rate of 3 ml kg^-1^ h^-1^.

The patient’s lungs will be ventilated with the GE Aisys Carestation™ using volume-controlled ventilation with square-wave flow as follow: VT of 8 ml kg^-1^ of predicted body weight (PBW), inspiratory-to-expiratory ratio of 1:2 with an end-inspiratory pause of 10%, PEEP of 5 cmH_2_O, inspiratory oxygen fraction (FiO_2_) of 0.5 and respiratory rate (RR) adjusted to maintain end-tidal CO_2_ (EtCO_2_) between 35–55 mmHg.

*Monitoring*

To monitor respiratory mechanics and efficiency the NICO monitor and the software DataColl (both Respironics, Wallingford, CT) will recorde respiratory, SpO_2_, and VCap data. Respiratory data will consisted of VT, airway and plateau pressures (Paw and Pplat), dynamic respiratory compliance (Cdyn = VT/ peak pressure − PEEP), expiratory airway resistance (Raw = peak pressure − PEEP/flow) and driving pressure (Pplat – PEEP).

The mainstream CO_2_ sensor will be zeroed and placed at the airway opening. Expired CO_2_ concentrations and VT data will be downloaded into MATLAB® (MathWorks, Natick, MA), which constructs volumetric capnograms according to the functional approximation using the Levenberg–Marquardt algorithm. We measured (1) VTCO_2_,br or tidal elimination of CO_2_ within one breath measured by integrating the area under the curve (AUC) of Vcaps, (2) the normalized VTCO_2_,br to alveolar tidal volume as follow:

VTCO_2_,br / VTalv

(3) dead space, calculated noninvasively using Bohr’s formula:

VDBohr (VD/VT) = (PACO_2_−PēCO_2_)/PACO_2_

where PACO_2_ is the mean alveolar partial pressure of CO_2_ measured at the midpoint of the alveolar plateau phase III and PēCO_2_ is the mixed expired partial pressure of CO_2_, (4) VDalv/VTalv calculated as follow:

VDalv/VTalv = (VDPhy – VDaw) = [(VDBohr/VT) x VT – VDaw] / VTalv.

*Experimental protocol*

After anesthesia stabilization, baseline data will be recorded during 10 minutes. The first 5 minutes will be considered for stabilization and the last 5 min of each VT will be analyzed. Five cmH_2_O of PEEP will be maintained during this part of the study.

Durign the next step of the protocol a recruitment maneuver will be performed as previously described^9^. At this moment patients will be randomized to continue with 5 cmH_2_O of PEEP after the RM (n = 16) or either to received an open–lung PEEP determined by a decremental PEEP titration trial (n = 16). This PEEP trial consists in a decrement in PEEP in step of 2 cmH_2_O during 2 minutes, from 20 to zero, looking for the highest dynamic compliance, which will consider as the optimal PEEP^10^. Thereafter, a new alveolar recruitment maneuver will be performed as described above to re-open alveoli collapsed during the decremental PEEP titration. The OL-PEEP (optimal PEEP) will be applied and maintained throughout the study period.

*Statistical analysis*

A total of 36 patients will be included in the study. First, the patient baseline variables will be described and the homogeneity of these groups evaluated using appropriate statistical tests for the type of variable being analyzed (mean difference of proportions, chi-square, ANOVA, with a corresponding confidence interval of 95%). The parameters will be presented as mean (± SD) or median (interquartil range). Statistical analysis will be performed using the SPSS 20.0 software package (SPSS, Chicago, IL, USA). P-values < 0,05 are considered significant.

1. 1 El efecto del tamaño de la muestra es la cuantificación de la magnitud de la diferencia. Detalla el tamaño de una diferencia (por ejemplo, entre dos valores medios) que debe considerarse como de interés, es decir, la magnitud que debe tener una diferencia para considerarla como significativa. En otras palabras, afinando con el efecto del tamaño de la muestra, podemos detectar diferencias (por ejemplo, entre dos valores medios) a nivel de décimas, o de centésimas. Cuanto más exigente sea nuestra necesidad (detectar las diferencias en las centésimas, que no en las décimas), el tamaño de la muestra será mayor. En nuestro caso, se ha optado por un efecto del tamaño de la muestra igual a 0,25; un efecto del tamaño medio, ni muy poco exigente, ni muy exigente (Quinn and Keough, 2002). [↑](#footnote-ref-1)
2. 2 El nivel de significatividad de un test se define como la probabilidad de tomar la decisión de rechazar la hipótesis nula (H_0_) cuando ésta es verdadera (decisión conocida como Error tipo I, o "falso positivo"). En nuestro caso, se ha tomado un nivel de significación estándar de 0,05 (Casella and Berger, 2002). [↑](#footnote-ref-2)
3. 3 La potencia del test mide la confianza que tenemos para poder detectar diferencias (por ejemplo entre valores medios) si es que existen. En nuestro caso, trabajamos con un 95% se confianza (Casella and Berger, 2002). [↑](#footnote-ref-3)
